# Comparison of Soil Bioconditioners and Standard Fertilization in Terms of the Impact on Yield and Vitality of *Lolium Perenne* and Soil Biological Properties

**DOI:** 10.1515/biol-2019-0076

**Published:** 2019-12-31

**Authors:** Dorota Swędrzyńska, Waldemar Zielewicz, Arkadiusz Swędrzyński

**Affiliations:** 1 Department of General and Environmental Microbiology, Poznań University of Life Sciences, 60 656 Poznań, Poland; 2 Department of Grassland and Natural Landscape Sciences, Poznań University of Life Sciences, 60 632 Poznań, Poland

**Keywords:** chlorophyll, enzymatic activity of soil, perennial ryegrass, soil microorganisms

## Abstract

The aim of the study was to compare the influence of selected soil bioconditioners and traditional fertilisation P+K+Ca on the vitality and yield of *Lolium perenne* and on the microbiological state of soil. The study was conducted between 2008 and 2009 – it was based on a field experiment started in 2006, in Brody, at the Agricultural Experiment Station of the Poznań University of Life Sciences, Poland. The factorial experiment was conducted in a randomized block design, with three replications. Two experimental factors were used: - non-nitrogen fertilisation (Physio-Mescal G18, PRP-SOL, Effective Microorganisms, Effective Microorganisms + Ca, P+K+Ca); - nitrogen fertilisation (N0 and N200 kg ha^-1^). The following parameters were measured: the yield of dry matter of perennial ryegrass, the plants’ vitality (chlorophyll concentration), the count of selected groups of soil microorganisms (heterotrophic, oligotrophic, copiotrophic bacteria, actinobacteria, fungi), soil enzymatic activity (dehydrogenases, acid phosphatase), and soil pH. The experiment showed that the bioconditioners were not an alternative to traditional mineral fertilisation, especially to nitrogen fertilisation, as a basic yield factor, but they could be a very valuable supplement to this fertilisation, and help to maintain the right biological potential of soil and its fertility, especially in the places where no manure or other non-chemical fertilisers are used.

## 1 Introduction

An alternative to traditional mineral fertilization – especially in conditions of sustainable or ecological agriculture – is the use of so-called biofertilizers or soil bioconditioners. The purpose of these preparations is to improve the use of soil production potential and fertility by stimulating the microbiological availability of nutrients and other mechanisms of beneficial effects of microorganisms on the growth and development of plants. In recent years, numerous preparations such as: PRP-SOL, Physio-Mescal G18 or EM (Effective Microorganisms) have appeared at the Polish market.

The product PRP-SOL is a mineral fertilizer with a positive effect on a number of soil parameters, the use of which leads to an increase in soil’s fertility [1]. It is produced on the basis of calcium carbonate contains magnesium and sodium enriched with and 48 trace elements [2] having important functions in plants and microbial cells [3].

Another innovative fertilizer preparation that has obtained the status of ecological fertilizer is Physio-Mescal G 18. This fertilizer improves the soil structure, stabilizes its reaction and increases the enzymatic activity of soil microorganisms. In addition, it stimulates the development of the root system of plants and, consequently, positively influences the absorption and intake of nutrients by plants [4, 5].

In recent years, Effective Microorganisms (EM) microbiological preparation [6] has been more and more often used in sustainable and ecological agriculture. The preparation is a combination of about 80 taxa of microorganisms. The most important species are lactic bacteria (*Lactobacillus casei*, *Streptococcus lactis*), photosynthesis bacteria (*Rhodopseudomonas palustris*, *Rhodobacter spae*), yeast (*Saccharomyces albus*, *Candida utilis*), actinobacteria (*Streptomyces albus, S. griseus*) and molds (*Aspergillus oryzae*, *Mucor hiemalis*) [7, 8]. The most frequently mentioned positive effects of this preparation are: increasing soil biological activity, increasing the content of humus, reducing the occurrence of plant pathogens as well as increasing the quantity and improving the quality of crops [7].

Fertilization determines the size and quality of crop yields but is also one of the main factors of agricultural influence on soil environment. Among primary indicators of soil environment condition and soil fertility are its biological parameters such as microbiological biomass content and number of taxonomical-ecological groups of soil microorganisms such as heterotrophic, copiotrophic or oligotrophic bacteria, actinobacteria, fungi et al [9, 10].

The presence and functioning of soil microorganisms are related to soil enzymatic activity - among others, the activity of dehydrogenases and phosphatases [11, 12]. It is widely believed that the enzymatic activity of soil in connection with its selected chemical and physical properties reflects its fertility and intensity of processes occurring in it [11, 12, 13, 14].

The aim of the study was to compare the effect of selected soil bioconditioners (Physio-Mescal G18, PRP-SOL, Effective Microorganisms) and standard fertilization on the viability and yield of perennial ryegrass (*Lolium perenne* L.) and a microbiological status of the soil.

## 2 Materials and methods

The research was carried out over two growing seasons in the years 2008-2009 and based on field experience founded in 2006, in Brody (52°26’ N; 16°17’ E), at Agricultural Experimental Station of Poznan University of Life Sciences, Poland.

The field experiment was established on soil developed from loamy sand, underlain by sandy loam and classified as Albic Luvisol [15]. The content of available nutrients in the topsoil, measured each year before fertilization, was high for phosphorus - 82 mg P kg^-1^ soil, medium for potassium - 138 mg K kg^-1^ soil (double lactate method), and medium for magnesium - 59 mg Mg kg^-1^ soil (Schachtschabel method).

The experiment was established in a randomized block design, in three replications on plots of 24m^2^. The effect of using soil bioconditioners and standard fertilization in the cultivation of perennial ryegrass (*Lolium perenne* L.), variety “Anna” on chlorophyll concentration and crop yield and on soil biological state, expressed by the number of selected groups of soil microorganisms and their enzymatic activity was investigated. The ryegrass was sown at 35 kg ha^-1^. The forecrop for ryegrass was winter rape. Two experimental factors were used: - non-nitrogen fertilization (control, P+K+Ca; EM-1+Ca; EM-1; Physio-Mescal G18; PRP-SOL), - fertilization with nitrogen (0 kg N ha^-1^ and 200 kg N ha^-1^). A total of 12 experimental combinations were obtained. The following doses of bioconditioners and standard fertilizers were used:

– Physio -Mescal G18 (18% P_2_O_5_, 65% CaCO_3_, 5% MgO, brown algae extract - Physio+) - 450 kg ha^-1^,
– PRP SOL (30% CaO, 8% MgO, 3,5% Na and microelements) - 200 kg ha^-1^,
– EM-1 – 1 dm^3^ EM-1 + 100 l H_2_O ha^-1^,
– EM-1+Ca - 1 dm^3^ EM-1 + 100 l H_2_O ha^-1^ + 100 kg CaCO_3_ ha^-1^,
– traditional mineral fertilization P+K+Ca (triple superphosphate + potassium salt + calcium carbonate fertilizer) - 80kg P_2_O_5_ ha^-1^ + 80kg K_2_O ha^-1^ + 100kg CaCO_3_ ha^-1^ (once in spring),
– nitrogen fertilization (ammonium sulphate) -200kg N ha^-1^ (50kg N ha^-1^ per regrowth).

Weather conditions prevailing in the vegetation season of both study years were varied (Table 1). In the first year (2008), the weather was very variable. The sum of precipitation was high, but they were very unevenly distributed. A relatively cool and humid April was followed by warm and very dry May and June, and hot July. Thus, the conditions during the second regrowth of the ryegrass green growth were particularly unfavorable and delayed its harvest. After a favorable August vegetation, characterized by moderate air temperatures and very high rainfall, there was a dry September. The harvest of the third regrowth of ryegrass was also late. However, it was followed by optimal temperatures and precipitation in October, which allowed the harvest of the fourth regrowth of the green growth. In the second year of research (2009), moderate and adequate air temperatures prevailed, and precipitation, although significantly lower than a year earlier, was evenly distributed in individual months. Trends observed in the first year of research appear more frequently and regularly and become a permanent element of climate change observed in Wielkopolska, as demonstrated by Goliński et al. [16] in their study.

**Table 1:** Weather conditions during the vegetation period of perennial ryegrass in RGD Brody in the years 2008 and 2009

Month	Average air temperature (°C)		Total rainfal (mm)	
	
	2008	2009	2008	2009
III	4.2	4.3	75.7	13.3
IV	8.7	11.7	120.7	85.3
V	15.2	13.4	19.5	79.3
VI	19.1	15.7	8.6	68.1
VII	20.0	19.7	80.1	31.4
VIII	18.8	19.7	171.5	50.0
IX	13.9	15.6	29.8	73.9
X	10.0	7.9	74.9	45.4
Average temperature	13.7	13.5	**-**	**-**
Total rainfall	**-**	**-**	580.8	446.7

The analysis of plant material consisted in determining the DM yield of the sward for each of four regrowths of ryegrass harvested each year, and the determination of plant viability. The dry matter yield was determined by the dryer-weight method based on the experimental mowing from the surface of 7.5 m^2^ for each plot. In 2008, the 1^st^ regrowth was collected on May 19^th^, 2^nd^ – July 29^th^, 3^rd^ – September 29^th^ and 4^th^ – October 14^th^. In 2009, harvests were held on: the 1^st^ regrowth – May 14^th^, 2^nd^ – July 8^th^, 3^rd^ – August 20^th^ and 4^th^ – September 28^th^.

The chlorophyll concentration of the ryegrass was evaluated immediately before the collection of subsequent green growth regrowth. With the help of the Hydro N-tester, the so-called green leaf index was determined, expressed in the value of SPAD (soil plant analyses development). SPAD is very strongly correlated with the content of chlorophyll (a + b), whose concentration in leaf blades is considered a reliable indicator of plant viability [17, 18]. The determinations were made on the 30 youngest yet fully formed leaf blades.

Soil samples for microbiological analyzes and pH (in H_2_O) determination came from the turf zone of the perennial ryegrass (0-15 cm). The soil was collected with Egner’s staff in four terms designated by a set of successive green growth regrowth harvests.

The number of microbial populations in the soil was analyzed by counting the CFU (colony forming units) of heterotrophic, oligotrophic and copiotrophic bacteria, actinobacteria and fungi using specific culture media in Petri dishes. For heterotrophic bacteria (counted after 56 days of incubation at the temperature of 25°C) a ready-to-use Merck-Standard count agar medium (3g yeast extract; 5.0g peptone from casein; 5g sodium chloride; 12g agar, 1dm^3^ H_2_O) was used [19]. Oligotrophic bacteria were counted on diluted nutritive broth medium (0.1g peptone, 0.1g beef extract, 0.05g sodium chloride, 20g agar, 1dm^3^ H_2_O) after 21 incubation days at 28°C [20]. Copiotrophic bacteria were determined on nutritive broth medium (10g peptone, 10g beef extract, 5g sodium chloride, 20g agar, 1dm^3^ H_2_O) after 7 days of incubation at 28°C [20]. For fungi Martin’s nourishing substrate (1g KH_2_PO_4_, 0.5g MgSO_4_, 5g peptone, 10g glucose, 3.3 ml Bengal, 0.1g chlortetracycline, 25g agar, 1dm^3^ H_2_O) was used (counted after 5 days of incubation at 24°C) [21]. Actinobacteria were assessed on Pochon substrate (0.05g asparagine, 0.1g nystatin, 2g starch, 5g K_2_HPO_4_, 2.5g MgSO_4_·7H_2_O, 2.5g NaCl, 0.05g MnSO_4_·5H_2_O, 0.05 g Fe_2_(SO_4_)_2_·5H_2_O, 25g agar, 1dm^3^ H_2_O), following incubation for 7 days at the temperature of 26°C [22]. The mean number of colonies was converted into soil dry matter on the basis of used dilution of soil solution and moisture of the soil sample.

Soil enzymatic activity was also determined in conditions of different fertilization. The performed examination of this activity was based on the determination of the activities of dehydrogenases (DHA) and acid phosphatase (PHOS-H). The activity of dehydrogenases (EC 1.1.1.) was identified by spectrophotometric method, using as substrate 1% TTC (2,3,5- triphenyltetrazolinum chloride) [23]. The activity of acid phosphatase (EC 3.1.3.) was determined using as substrate p-nitrophenylophosphate sodium [24].

The results were tested by using standard variance analysis (ANOVA) for the randomised complete block design. Mean separations were made for significant effects with LSD and Tukey tests at the probability of α ≤ 0.05. All statistical analyses were carried out with the program Statistica 7.1 software.

## 3 Results and discussion

The effect of fertilizer combinations on the yield of perennial ryegrass is presented in Tables 2 and 3, separately for each year.

**Table 2:** Influence of the fertilizer combinations on the yield of perennial ryegrass sward in 2008 - first year of study (dt DM ha^-1^)

Experimental combination	1^st^ regrowth	2^nd^ regrowth	3^rd^ regrowth	4^th^ regrowth	Sum
Control	10.1 c	2.4 c	13.2 c	3.3 b	29.0 c
P+K+Ca	15.2 b	3.6 b	18.7 b	4.4 a	41.9 b
EM-1	11.3 c	2.6 c	12.4 d	3.0 b	29.3 c
EM-1+Ca	17.3 a	4.2 ab	18.2 b	3.6 ab	43.3 ab
Physio- Mescal	18.7 a	4.7 a	19.4 a	4.2 a	47.0 a
PRP- SOL	17.6 a	4.4 a	18.7 b	3.9 ab	44.6 ab

Average	15.0	3.6	16.7	3.7	39.1

LSD_α=0.05_	1.90	0.51	0.61	0.75	4.99

Control+N	37.1 f	8.1 d	18.3 d	13.3 d	76.8 d
P+K+Ca+N	45.2 a	11.2 a	23.1 b	17.4 a	96.9 a
EM-1+N	37.4 e	8.4 d	18.1 d	13.1 d	77.0 d
EM-1+Ca+N	42.6 d	9.6 c	22.4 c	15.2 c	89.8 c
Physio- Mescal +N	44.3 b	10.6 b	25.8 a	17.7 a	98.4 a
PRP-SOL+N	43.1 c	9.3 c	22.7 bc	16.5 b	91.6 b

Average	41.6	9.5	21.7	15.5	88.4

LSD_α=0.05_	0.23	0.36	0.45	0.55	1.53

Means followed by the same letters do not differ significantly (p=0.05)

**Table 3:** Influence of the fertilizer combinations on the yield of perennial ryegrass sward in 2009 - second year of study (dt DM ha^-1^)

Experimental combination	1^st^ regrowth	2^nd^ regrowth	3^rd^ regrowth	4^th^ regrowth	Sum
Control	10.2 cd	6.3 d	2.1 c	1.6 b	20.2 e
P+K+Ca	14.2 a	11.7 a	3.4 a	2.6 a	31.9 a
EM-1	9.8 d	6.1 d	2.3 c	1.7 b	19.9 e
EM-1+Ca	10.4 cd	8.1 c	2.6 bc	2.2 ab	23.3 d
Physio- Mescal	12.1 b	10.6 b	2.9 b	2.4 a	28.0 b
PRP-SOL	10.7 c	9.7 b	2.7 bc	2.2 ab	25.3 c

Average	11.2	8.7	2.6	2.1	24.7

LSD_α=0.05_	0.61	0.91	0.35	0.66	1.83

Control+N	49.4 d	30.9 e	8.6 c	7.2 e	96.1 e
P+K+Ca+N	61.7 a	40.2 a	16.1 a	13.6 a	131.6 a
EM-1+N	49.7 d	31.1 e	8.9 c	7.6 e	97.3 e
EM-1+Ca+N	52.3 c	32.0 d	9.6 c	8.8 d	102.7 d
Physio-Mescal+N	61.2 a	38.5 c	13.5 b	12.7 b	125.9 b
PRP-SOL+N	59.4 b	39.2 b	12.6 b	11.8 c	123.0 c

Average	55.6	35.3	11.5	10.2	112.7

LSD_α=0.05_	1.20	0.59	1.36	0.42	1.94

Means followed by the same letters do not differ significantly (p=0.05)

Yields of ryegrass were strongly varied in both years of research (second and third year of ryegrass use), in regrowths and in experimental combinations. In the first year of research (Table 2) the average yield of perennial ryegrass was 39.1 dt DM ha^-1^ without N fertilization, and 88.4 dt DM ha^-1^ in combinations with N fertilization. The difference was more than double. Nitrogen fertilization also modified the share of yields of individual regrowths in the whole year yield. In combinations without nitrogen fertilization, the yield of the first regrowth accounted for 38.3% of total yield, the second – 9.2%, the third – 43% and the fourth – only 9.4%. In combination with N fertilization, the share of the first regrowth in the total yield was by far the largest (as much as 47%) and the second regrowth – the lowest (only 10.6%.). Yields of the third and fourth regrowths accounted for 24.5% and 17.5% of the harvest collected throughout the vegetation season, respectively.

The lowest yield of the second regrowth in all fertilizer combinations was definitely determined by the strong drought prevailing in its vegetation season. In Wielkopolska, climate change contributes to the regular reduction of grassland sward yields due to the increase in air temperature in June and August and annual air temperature [16].

The most important issue is the impact of soil bioconditioners and traditional P+K+Ca fertilization on the yielding of perennial ryegrass. It turned out that the fertilization with Physio-Mescal G18 was the most effective for both the yields of individual regrowths and the annual yield. The use of this preparation translated into a year-round yield of 47.0 dt of ryegrass green growth dm per 1 ha in combination with no nitrogen fertilization and 98.4 dt dm ha^-1^ under the conditions of additional nitrogen fertilization. In the absence of nitrogen fertilization, few (statistically insignificant) lower yields were obtained with EM-1 + Ca (43.3 dt dm ha^-1^) and PRP- SOL (44.6 dt dm ha^-1^), while in combinations with nitrogen fertilization equivalent yields were obtained with traditional P + K + Ca fertilization. The lowest yields, regardless of nitrogen fertilization, were obtained in control combinations and in combinations with the use of EM-1 without the addition of Ca.

In the second year (Table 3), differences in the average total yield between combinations not fertilized with nitrogen (only 24.7 dt dm ha^-1^), and those fertilized with this element (112.7 dt dm ha^-1^) were even larger – almost five times. Similar proportions were recorded in individual regrowths. The distribution of the share of regrowths in relation to the whole-year yield was also similar in both variants of nitrogen fertilization. In the nitrogen-free combinations, the yields of the first and second regrowth accounted for 45% and 35% of the annual yield respectively, and the third and fourth yields - 11.5 and 8.5%. In the nitrogen-fertilized combinations, the share of subsequent regrowths in the annual yield structure was: 49.3%, 31.3%, 10.2% and 9.0%.

The fertilizer combination that had the most favorable effect on the yield of perennial ryegrass in the second year of research was P+K+Ca fertilization. This fertilization resulted in significantly higher yields of ryegrass dry matter, both in the variant without additional nitrogen fertilization (31.9 dt ha^-1^), and with its application (131.6 dt ha^-1^). Similarly, to the previous year, the use of EM-1 proved to be the least effective – the yield did not differ significantly from the control, regardless of nitrogen fertilization.

Nitrogen fertilization turns out to be decisive in terms of the yield of perennial ryegrass green growth. According to many authors, the effectiveness of nitrogen fertilization of agricultural crops [25] should be expressed not only by the quantitative and qualitative changes of the useful yield, but also with the help of other measures, for example agricultural efficiency (increase in yield per N unit used in fertilizers) or physiological efficiency. Based on the analysis of variance, it can be stated that the influence of the applied experimental factor of various fertilization combinations on the yield of perennial ryegrass was significant. On the basis of the obtained results of yielding of individual regrowths, it can be noticed that the use of the applied doses of nitrogen fertilization of 50 kg N ha^-1^ for each regrowth depended on the vegetation season and the distribution and amount of precipitation, which was especially visible in 2008.

Beneficial effects of application of calcium fertilizers such as Soleflor in fertilizer combinations on the increase in the acquired yields of orchard grass green growth were noted by some autors, e.g. [26]. In the first year of the study, both combinations with Soleflor gave higher yields of the green growth than NPK control. It was also reported that the higher the dose of this fertilizer, the higher the yield of the green growth. In the case of the Soleflor 300 combination, an increase in yield by 6% was found, and in combination with a double dose of this fertilizer – Soleflor 600, an increase in the yield of green growth by 12% was found in relation to NPK control. The satisfactory effects of using calcium fertilizers such as Physiomax 975 and Physio-Mescal G 18 were also noted in the cultivation of lucerne [5]. In turn, Soleflor fertilizer used in sowing the *Fabaceae* and grass mixture positively influenced higher percentage and durability in the Timothy grass and white clover green growths compared to the absolute control and combinations with only standard NPK fertilization [27]. Due to the composition of the Soleflor fertilizer used by these authors as well as the fertilizers PRP- SOL and Physio Mescal G18 used in this experiment, which also contain calcium carbonate in their composition, a positive effect on the soil pH increase, better use of phosphorus and potassium by plants and deactivation of harmful effects of aluminum on plants can be expected in the subsequent years of application. The procedure of soil liming is important especially in preventing the progressive acidification of soil – one of the reasons for which is too intense nitrogen fertilization [20].

**Figure 1:**
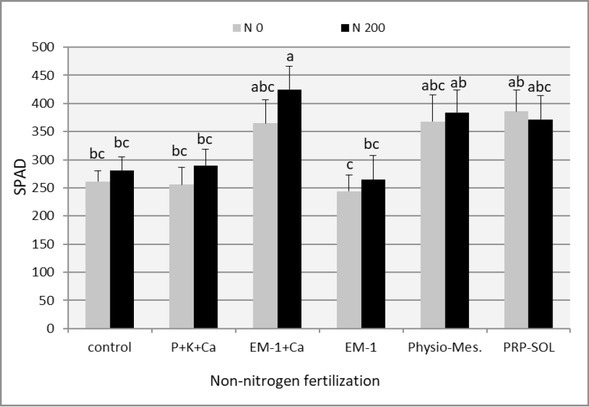
The effect of fertilizer combinations on the chlorophyll content in perennial ryegrass leaf blades (mean 2008-2009). Means followed by the same letters do not differ significantly (p=0.05)

The content of chlorophyll in leaf blades of perennial ryegrass (Table 4, Figure 1), expressed as the SPAD value, was the highest in the first yield, i.e. in May, and was 376.8 on average for all combinations without nitrogen fertilization, and 389.9 – for combinations with nitrogen fertilization. In the last, (the 4^th^) regrowth of the perennial ryegrass green growth, the lowest content of chlorophyll in leaf blades of ryegrass was found (SPAD value: 234.0 on average in combinations without nitrogen fertilization and 245.8 in combinations fertilized with nitrogen). This distribution of chlorophyll in leaf blades of fodder grasses during the vegetation season is typical. In the first regrowth, the development of grasses occurs most often in the best weather conditions for them, which translates into a longer plant life. In the autumn, at the end of the vegetation and when plants metabolism is slowed down, the chlorophyll content in the leaves of grasses decreases sharply [17]. The beneficial effect of nitrogen fertilization on the content of chlorophyll is, in turn, widely known and would not require commentary, if it were not for the fact that the differences between fertilized and non-fertilized nitrogen combinations turned out to be small and statistically insignificant.

**Table 4:** Influence of the fertilizer combinations on the chlorophyll content (SPAD index) in perennial ryegrass leaf blades over the vegetation period (mean 2008- 2009)

Regrowth	Biofertilization (A)	N-fertilization (B) (kg^.^ ha^-1^)		Average
		0	200	
I	Control	257.5	264.0	260.7
	P+K+Ca	294.8	310.5	302.6
	EM-1+Ca	483.0	493.3	488.1
	EM-1	261.3	303.8	282.5
	Physio- Mescal	490.8	474.3	482.5
	PRP -SOL	473.8	493.3	483.5

	Average	376.8	389.9	383.4

LSD_α= 0,05_ A				39.7
LSD_α= 0,05_ B				ns
LSD_α= 0,05_ AxB				ns

II	Control	267.5	337.0	302.3
	P+K+Ca	269.0	294.0	281.5
	EM-1+Ca	338.0	450.5	394.3
	EM-1	258.0	279.0	268.5
	Physio- Mescal	348.0	375.0	361.5
	PRP -SOL	374.5	373.0	373.8

	Average	309.2	351.4	330.3

LSD_α= 0,05_ A				38.4
LSD_α= 0,05_ B				ns
LSD_α= 0,05_ AxB				ns

III	Control	286.5	303.0	294.8
	P+K+Ca	272.0	314.5	293.3
	EM-1+Ca	383.5	451.5	417.5
	EM-1	263.0	282.0	272.5
	Physio- Mescal	385.0	407.5	396.3
	PRP-SOL	409.5	386.0	397.8

	Average	333.2	357.4	345.4

LSD_α= 0,05_ A				69.09
LSD_α= 0,05_ B				ns
LSD_α= 0,05_ AxB				ns

IV	Control	233.0	218.5	225.8
	P+K+Ca	187.0	241.5	214.3
	EM-1+Ca	255.5	306.0	280.8
	EM-1	194.5	195.0	194.8
	Physio- Mescal	248.5	280.5	264.5
	PRP- SOL	285.5	233.5	259.5

	Average	234.0	245.8	240.0

LSD_α= 0,05_ A				ns
LSD_α= 0,05_ B				ns
LSD_α= 0,05_ AxB				ns

ns -not significant

The dependences presented above for individual regrowths are reflected in the mean values ​from the whole vegetative period (Figure 1). In this approach, the highest viability of perennial ryegrass was observed after the application of the EM + Ca preparation. The difference in the SPAD values in combinations without nitrogen fertilization, as well as with additional fertilization with this element, was respectively 44% and 46.5% in comparison to traditional P + K + Ca fertilization. The use of Physio-Mescal G18 and PRP-SOL was similar.

In this context, the influence of soil bioconditioners on the content of chlorophyll in leaf blades of the perennial ryegrass is interesting. Differences between levels were greater than in the case of nitrogen fertilization and, with the exception of the fourth regrowth, statistically significant. As in the case of yields, most of the regrowths and regardless of nitrogen fertilization, the EM-1 preparation was the least effective. Its effect on plant viability was usually comparable to the control combination. P+K+Ca fertilization also did not significantly influence the concentration of chlorophyll in the ryegrass leaves. On the other hand, Physio-Mescal G18 and PRP-SOL preparations, as well as EM-1 used together with calcium carbonate, had a clearly positive and statistically significant effect on this feature. The use of these preparations in the case of the majority of regrowth, contributed significantly to the increase in chlorophyll content in leaf blades of ryegrass, both in combinations not fertilized with nitrogen and in the fertilized ones.

In the literature on the subject, there are many examples of positive effects of the use of soil improvers or bioconditioners on the development, protection, viability and yielding of crop plants, e.g. [29, 30]. However, there are also many examples of the lack of such impact [6]. The authors of this work also noticed a beneficial effect on the vitality of plants in relation to the Soleflor soil bioconditioner in the cultivation of perennial ryegrass [31], in the *Fabaceae* and grass mixture [27] and in the case of orchard grass [26]. In another study, fertilization of Lucerne with Physio-Mescal G 18 and Physiomax 975 resulted in a much worse effect than traditional mineral fertilization with superphosphate and potassium salt [5].

**Figure 2:**
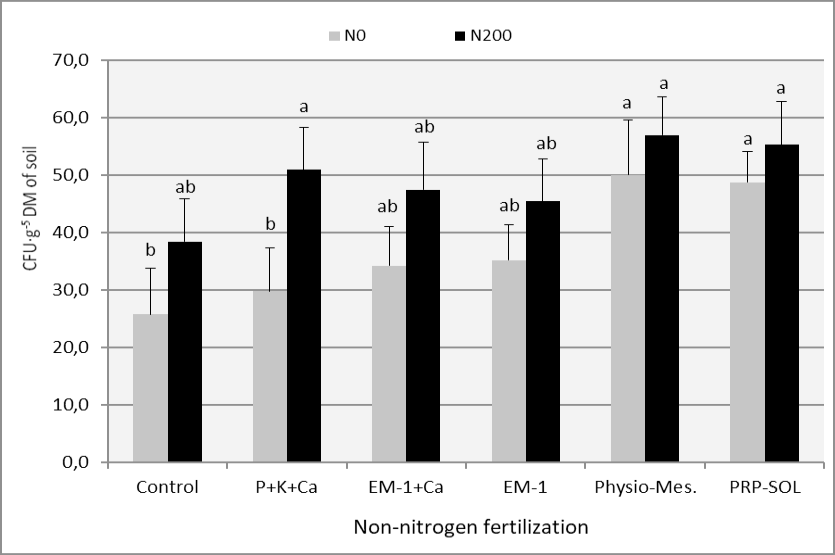
The effect of fertilizer combinations on the oligotrophic bacteria number in the soil (mean 2008-2009). Means followed by the same letters do not differ significantly (p=0.05)

**Figure 3:**
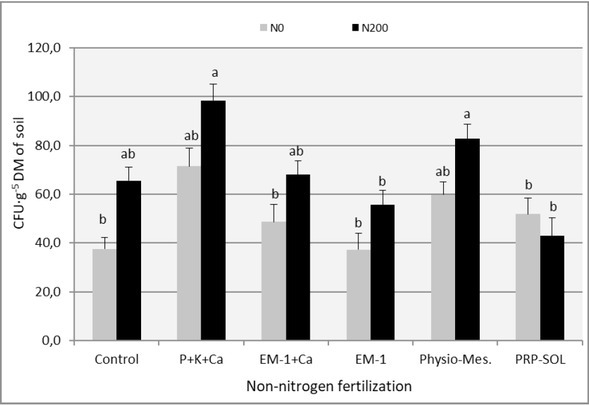
The effect of fertilizer combinations on the copiotrophic bacteria number in the soil (mean 2008-2009). Means followed by the same letters do not differ significantly (p=0.05)

**Figure 4:**
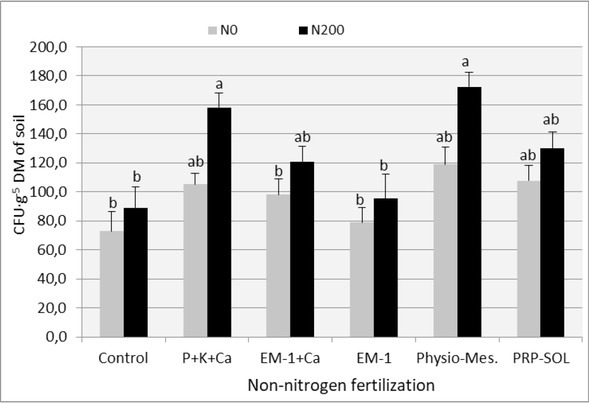
The effect of fertilizer combinations on the actinobacteria number in the soil (mean 2008-2009). Means followed by the same letters do not differ significantly (p=0.05)

The results of the analysis of the impact of the experimental combinations used on the population size of selected groups of soil microorganisms (oligotrophic bacteria, copiotrophic bacteria, actinobacteria) are presented in Table 5-7 and in Figure 2-4. The number of heterotrophic bacteria and the number of fungi was not included in these lists, as the variability within the obtained results from this range prevented their correct and unambiguous interpretation.

**Table 5:** Influence of the fertilizer combinations on the number of oligotrophic bacteria in soil over the vegetation period

Non-nitrogen fertilization (A)	Oligotrophic bacteria (CFU·g^-5^ DM of soil)					
		2008			2009	

			Nitrogen fertilization (B) (kg N^.^ha^-1^)			
	
	0	200	Average	0	200	Average
1^st^ regrowth						

Control	6.50	9.54	8.02	23.01	31.59	27.30
P+K+Ca	6.46	8.38	7.42	28.22	94.73	61.48
EM-1+Ca	8.40	4.78	6.59	56.94	65.33	61.13
EM-1	9.74	19.10	14.42	69.16	61.60	65.38
Physio- Mescal	6.45	17.27	11.86	44.46	82.68	63.57
PRP-SOL	6.39	8.54	7.47	86.63	57.06	71.85
Average	7.32	11.27	9.30	51.40	65.50	58.45
LSD _α=0.05_ A		6.71			13.27	
LSD _α=0.05_ B		2.14			ns	
LSD_α=0.05_ AxB		ns			18.76	

2^nd^ regrowth						

Control	7.14	16.26	11.70	43.53	58.33	50.93
P+K+Ca	9.49	14.88	12.19	41.08	116.60	78.84
EM-1+Ca	10.93	21.29	16.11	56.05	127.93	92.00
EM-1	11.39	13.66	12.53	8.39	45.38	26.88
Physio- Mescal	9.93	15.08	12.51	141.35	132.56	136.96
PRP-SOL	7.86	11.94	9.90	113.33	92.33	102.85
Average	9.46	15.52	12.49	67.29	95.52	81.41
LSD _α=0.05_ A		ns			ns	
LSD _α=0.05_ B		ns			ns	
LSD_α=0.05_ AxB		ns			ns	

3^rd^ regrowth						

Control	44.61	54.33	49.47	26.73	28.46	27.60
P+K+Ca	39.27	68.41	53.84	45.62	11.21	28.42
EM-1+Ca	34.02	49.01	41.52	20.94	26.67	23.80
EM-1	48.24	79.49	63.87	25.16	26.60	25.88
Physio- Mescal	38.38	55.00	46.69	34.30	23.89	29.09
PRP-SOL	27.66	70.40	49.03	30.53	42.22	36.37

Average	38.70	62.77	50.74	30.55	26.51	28.53

LSD _α=0.05_ A		ns			ns	
LSD _α=0.05_ B		ns			ns	
LSD_α=0.05_ AxB		ns			ns	

4^th^ regrowth						

Control	23.66	46.45	35.06	31.05	62.10	46.57
P+K+Ca	41.61	71.08	56.35	26.36	21.66	24.01
EM-1+Ca	39.55	33.47	36.51	46.80	50.92	48.86
EM-1	65.40	74.29	69.85	44.08	43.32	43.70
Physio- Mescal	74.97	88.78	81.88	50.54	40.28	45.41
PRP-SOL	55.52	70.13	62.83	61.77	89.30	75.53

Average	50.12	64.03	57.08	43.43	51.26	47.35

LSD _α=0.05_ A		ns			ns	
LSD _α=0.05_ B		8.03			ns	
LSD_α=0.05_ AxB		11.31			ns	

ns -not significant

**Table 6:** Influence of the fertilizer combinations on the number of copiotrophic bacteria in soil over the vegetation period

Non-nitrogen fertilization (A)	Copiotrophic bacteria (CFU·g^-5^ DM of soil)					

		2008			2009	

	Nitrogen fertilization (B) (kg N^.^ha^-1^)					
	
	0	200	Average	0	200	Average
1^st^ regrowth						

Control	47.18	87.13	67.16	41.34	68.76	55.05
P+K+Ca	60.08	122.73	91.41	35.19	139.20	87.19
EM-1+Ca	61.80	96.48	79.14	37.05	45.54	41.29
EM-1	43.34	89.28	66.31	29.64	38.50	34.07
Physio- Mescal	34.79	82.58	58.69	90.44	58.65	74.54
PRP-SOL	38.88	59.22	49.05	42.94	27.10	35.02

Average	47.68	89.57	68.62	46.10	62.96	54.53

LSD _α=0.05_ A		35.03			26.23	
LSD _α=0.05_ B		41.49			10.06	
LSD_α=0.05_ AxB		54.49			50.60	

2^nd^ regrowth						

Control	25.41	32.71	29.06	2.30	67.71	35.00
P+K+Ca	32.01	45.50	38.75	122.85	162.72	142.78
EM-1+Ca	28.83	43.76	36.30	61.62	96.77	79.19
EM-1	26.53	28.37	27.45	5.75	28.37	17.06
Physio- Mescal	29.73	35.97	32.85	23.75	115.86	69.80
PRP-SOL	25.27	32.84	29.06	4.21	14.56	9.38

Average	27.96	36.53	32.24	36.74	81.0	58.87

LSD _α=0.05_ A		ns			81.79	
LSD _α=0.05_ B		ns			ns	
LSD_α=0.05_ AxB		ns			ns	

3^rd^ regrowth						

Control	62.57	119.89	91.23	29.52	36.05	32.78
P+K+Ca	86.96	146.30	116.63	37.80	16.76	27.28
EM-1+Ca	93.43	137.14	115.29	33.17	14.00	23.58
EM-1	75.44	71.09	73.27	26.88	41.69	34.29
Physio- Mescal	154.21	176.56	165.39	39.59	42.00	40.79
PRP-SOL	169.76	56.74	113.25	54.36	46.20	50.28

Average	107.06	117.95	112.51	36.88	32.78	34.58

LSD _α=0.05_ A		ns			11.13	
LSD _α=0.05_ B		ns			2.30	
LSD_α=0.05_ AxB		72.34			15.74	
4^th^ regrowth						
Control	62.45	79.94	71.20	30.28	32.58	31.43
P+K+Ca	115.71	143.25	129.48	80.94	11.40	46.17
EM-1+Ca	52.98	78.28	65.63	20.67	32.30	26.48
EM-1	60.51	108.10	84.31	30.40	39.90	35.15
Physio- Mescal	89.20	129.86	109.53	16.34	20.14	18.24
PRP-SOL	58.47	84.82	71.65	21.09	21.66	21.37

Average	73.22	104.04	88.63	33.28	26.33	29.81

LSD _α=0.05_ A		43.42			11.57	
LSD _α=0.05_ B		ns			ns	
LSD_α=0.05_ AxB		ns			16.36	

ns -not significant

**Table 7:** Influence of the fertilizer combinations on the number of actinobacteria in soil over the vegetation period

Non-nitrogen fertilization (A)	Actinobacteria number (CFU·g^-5^ DM of soil)					

		2008			2009	

	Nitrogen fertilization (B) (kg N^.^ha^-1^)					
	
	0	200	Average	0	200	Average
1^st^ regrowth						

Control	127.19	116.95	122.07	64.08	97.89	80.99
P+K+Ca	126.86	119.38	123.12	75.40	136.80	106.10
EM-1+Ca	86.22	105.37	95.80	99.48	171.73	135.60
EM-1	98.19	114.18	106.19	103.76	107.53	105.65
Physio- Mescal	123.08	114.97	119.03	96.62	212.00	154.31
PRP-SOL	98.26	121.38	109.82	87.38	159.07	123.23

Average	109.97	115.37	112.67	87.79	147.50	117.65

LSD _α=0.05_ A		ns			37.70	
LSD _α=0.05_ B		ns			43.20	
LSD_α=0.05_ AxB		30.21			57.47	

2^nd^ regrowth						

Control	67.04	83.34	75.19	71.3	123.58	97.44
P+K+Ca	138.44	184.25	161.35	148.20	160.83	154.52
EM-1+Ca	94.84	145.60	120.22	98.67	107.87	103.27
EM-1	66.21	74.68	70.45	59.36	112.31	85.84
Physio- Mescal	136.65	220.67	178.66	103.50	136.00	119.75
PRP-SOL	175.06	102.39	138.73	117.00	157.92	137.46

Average	113.04	135.16	124.10	99.67	133.09	116.38

LSD _α=0.05_ A		ns			56.86	
LSD _α=0.05_ B		ns			ns	
LSD_α=0.05_ AxB		98.91			80.41	

3^rd^ regrowth						

Control	53.04	52.97	53.01	79.20	149.80	114.50
P+K+Ca	30.97	33.68	32.33	134.46	238.43	186.45
EM-1+Ca	38.52	27.65	33.09	144.80	196.00	170.40
EM-1	78.04	57.66	67.85	71.33	89.04	80.19
Physio- Mescal	47.55	38.91	43.23	151.91	258.69	205.30
PRP-SOL	16.05	30.76	23.41	244.08	249.20	246.64

Average	44.03	40.27	42.15	154.96	179.53	167.25

LSD _α=0.05_ A		ns			58.08	
LSD _α=0.05_ B		ns			68.17	
LSD_α=0.05_ AxB		ns			82.15	

4^th^ regrowth						

Control	45.81	42.66	44.24	75.13	44.85	59.99
P+K+Ca	89.07	162.85	125.96	97.93	229.14	163.54
EM-1+Ca	121.66	111.84	116.75	100.62	101.70	101.16
EM-1	77.18	113.42	95.30	76.76	93.48	85.12
Physio- Mescal	163.86	181.40	172.63	129.20	215.40	172.30
PRP-SOL	93.07	142.03	117.55	31.64	78.28	54.96

Average	98.44	125.70	112.07	85.21	127.14	106.18

LSD _α=0.05_ A		14.21			58.79	
LSD _α=0.05_ B		12.45			ns	
LSD_α=0.05_ AxB		17.11			83.15	

ns -not significant

The number of microorganisms varied in individual regrowths. The strongest in 2008. The reason for the differences was variable soil moisture, differences in the development and condition of plants, and therefore in the amount of root secretions and arid plant debris.

Nitrogen fertilization increased the number of analyzed groups of the ryegrass turf microbiome in all experimental combinations. This impact was visible both in individual regrowths (Table 5-7) and in relation to the means from the entire study period (Figure 2-4). A dozen or even several dozen percent increase in the number of microorganisms under the influence of nitrogen fertilization is, however, not much when compared to the results obtained in the first year of use of ryegrass, presented in another paper by the authors [32]. The mechanism of influence of nitrogen fertilization on microorganisms is a modification of the C:N ratio and an increase in the nutrient substrate supply, which are root secretions and dead tissues of the plants which develop more intensively under these conditions [33, 34, 35, 36].

The analysis of the impact of individual biofertilizers on the amount of microorganisms in the soil based on averages from individual regrowths (Table 5-7) and from the entire vegetation period (Figure 2-4) makes it easy to notice that almost all fertilizer variants have influenced the increase in the number of investigated groups of microorganisms, especially oligotrophic bacteria and actinobacteria, This impact was not always statistically significant, though. The distinguishing combinations were the ones with Physio-Mescal G18 and with P+K+Ca fertilization, which boosted the content of almost all soil microbial groups in individual regrowths.

Some reports on the strong, beneficial effect on the soil microbiological balance of the EM-1 preparation, e.g. [37], have not been confirmed, though, and the obtained results confirm skeptical opinions about this preparation [6].

Its influence on the number of microorganisms was negligible and limited only to actinobacteria. No positive effect of the use of EM-1 with the simultaneous use of nitrogen fertilization was observed, which was visible in the first year of the application of ryegrass [32].

In contrast to the effect of nitrogen fertilization, non-nitrogen fertilization mainly stimulated the number of actinobacteria and oligotrophic bacteria, and these are ecological groups of microorganisms which most strongly indicate soil fertility [38].

**Figure 5:**
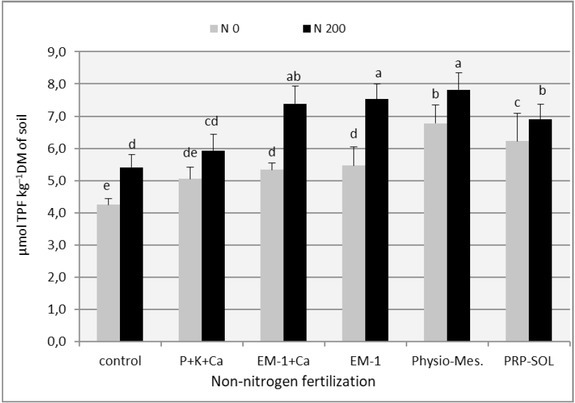
The effect of fertilizer combinations on the dehydrogenases activity in the soil (means from all vegetation period). Means followed by the same letters do not differ significantly (p=0.05)

**Figure 6:**
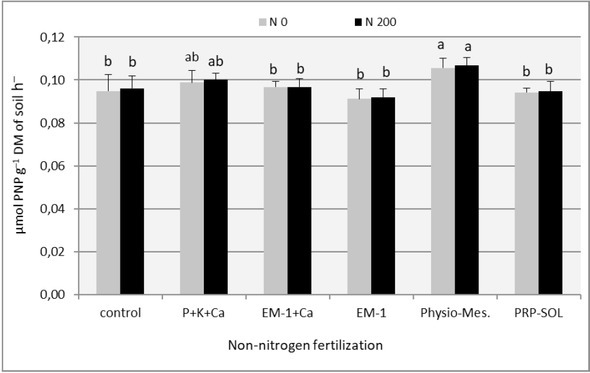
The effect of fertilizer combinations on the acid phosphatase activity in the soil (means from all vegetation period). Means followed by the same letters do not differ significantly (p=0.05)

The effect of fertilizer combinations on soil enzymatic activity (dehydrogenases and acid phosphatase) is presented in Table 8 and in Figure 5 and 6. The analysis of variance (p=0.05) showed that while in the first year of use of perennial ryegrass differences between experimental factors were almost always irrelevant, in the second year, the impact of studied factors on soil enzymatic activity – both in regrowth, as well as in relation to annual averages – was clear and statistically significant. In the first year, the enzymatic activity of the soil, in particular – the dehydrogenases activity, was very small, and with the applied research method, the results were difficult to interpret. It was probably due to strong drought prevailing during the vegetation season, which inhibited both plant vegetation and the activity of microbiological rhizosphere life. Hence, the results of soil enzymatic activity from 2008 were not included in the tabulations or in figures.

**Table 8:** Influence of the fertilizer combinations on the enzymatic activity of soil over the vegetation period

Non-nitrogen fertilization (A)	The enzymatic activity of soil					

	Dehydrogenases (μmol TPF·kg^-1^ DM of soil·24h^-1^)			Acid phosphatase (μmol PNP·g^-1^ DM of soil ·h^-1^)		

	Nitrogen fertilization (kg N^.^ha^-1^) (B)					

	0	200	Average	0	200	Average
1^st^ regrowth						

Control	8.30	6.60	7.45	0.0790	0.0869	0.0830
P+K+Ca	9.20	8.80	9.00	0.0859	0.0925	0.0892
EM-1+Ca	8.30	8.87	8.58	0.0887	0.0829	0.0858
EM-1	9.57	8.07	8.82	0.0837	0.0760	0.0799
Physio- Mescal	8.93	10.63	9.78	0.0943	0.0912	0.0928
PRP-SOL	9.10	9.83	9.47	0.0882	0.0797	0.0840

Average	8.90	8.80	8.85	0.0867	0.0849	0.0858

LSD _α=0.05_ A		0.301			0.0043	
LSD _α=0.05_ B		ns			Ns	
LSD_α=0.05_ AxB		0.420			0.0117	

2^nd^ regrowth						

Control	4.00	4.17	4.08	0.0802	0.0772	0.0787
P+K+Ca	4.77	4.73	4.75	0.0783	0.0802	0.0793
EM-1+Ca	6.00	6.80	6.40	0.0820	0.0850	0.0835
EM-1	4.13	6.97	5.55	0.0673	0.0796	0.0734
Physio- Mescal	8.33	6.96	7.65	0.1002	0.1015	0.1009
PRP-SOL	4.03	4.07	4.05	0.0815	0.0809	0.0812

Average	5.21	5.62	5.41	0.0816	0.0841	0.0828

LSD _α=0.05_ A		0.171			0.0180	
LSD _α=0.05_ B		ns			Ns	
LSD_α=0.05_ AxB		0.164			0.0062	

3^rd^ regrowth						

Control	3.33	7.30	5.32	0.1022	0.0993	0.1008
P+K+Ca	3.93	6.43	5.18	0.1051	0.1015	0.1033
EM-1+Ca	3.97	7.63	5.80	0.1040	0.0990	0.1015
EM-1	5.70	8.50	7.10	0.1039	0.0937	0.0988
Physio- Mescal	5.00	5.30	5.15	0.1072	0.0986	0.1029
PRP-SOL	5.83	7.13	6.48	0.0965	0.0956	0.0961

Average	4.63	7.05	5.84	0.1032	0.0980	0.1006

LSD _α=0.05_ A		0.382			0.0011	
LSD _α=0.05_ B		0.268			ns	
LSD_α=0.05_ AxB		0.586			0.0024	

4^th^ regrowth						

Control	1.37	3.60	2.48	0.1177	0.1211	0.1195
P+K+Ca	2.30	3.77	3.03	0.1261	0.1258	0.1260
EM-1+Ca	3.10	6.23	4.67	0.1120	0.1203	0.1162
EM-1	2.47	6.57	4.52	0.1100	0.1187	0.1144
Physio- Mescal	4.87	8.40	6.63	0.1207	0.1353	0.1280
PRP-SOL	5.93	6.57	6.25	0.1104	0.1226	0.1165

Average	3.34	5.86	4.60	0.1162	0.1240	0.1201

LSD _α=0.05_ A		0.431			0.0064	
LSD _α=0.05_ B		0.260			0.0050	
LSD_α=0.05_ AxB		0.410			0.0093	

ns -not significant

The average dehydrogenases activity was the highest in the first regrowth of the perennial ryegrass, and the lowest – in the second regrowth (Table 8). The use of nitrogen fertilization, significantly affected the activity of the dehydrogenases in the third and fourth regrowths. In principle, this statement applies to all experimental combinations and translates into average values over the entire growing season (Figure 5). The positive effect of nitrogen fertilization on dehydrogenases activity was in accordance with expectations, because this group of soil enzymes is considered one of the indicators of soil fertility, strongly correlated with it [39].

Also, the individual levels of non-nitrogen fertilization, indicated in Table 8 as factor A (biopreparations and P+K+Ca), significantly affected the dehydrogenases activity. However, in three out of four as well as the annual average, the activity of Physio- Mescal was the most effective. The effect of PRP-SOL and EM-1 and EM-1 + Ca preparations was less unambiguous, but also clear, stimulating dehydrogenases activity, especially in combination with nitrogen fertilization (Table 8, Figure 5). The influence of traditional fertilization (P+K+Ca) was much weaker over the entire vegetation season, however, also in this case the dehydrogenases activity was higher than in the control in most terms and in relation to the annual average.

Acid phosphatase activity in the soil under perennial ryegrass increased along the vegetation season. The lowest level was achieved in the first and second regrowths, and the highest – in the last one (Table 8, Figure 6). Nitrogen fertilization slightly modified the acid phosphatase activity – both in individual regrowths as well as in relation to the average of the entire vegetation season. Only in the last i.e. fourth regrowth, the phosphatase activity was slightly higher in the combinations fertilized with nitrogen, and the differences were statistically significant.

The effects of biopreparation fertilization in acid phosphatase were not as unambiguous as in the case of dehydrogenases. Most of the combinations did not differ significantly from the controls. Only the use of Physio-Mescal translated in a statistically significant way into higher acid phosphatase activity with respect to the annual average and most of the regrowths. P+K+Ca fertilization was in second place in this classification, however, differences in relation to control proved to be insignificant.

In this experiment, the effect of fertilization on the activity of soil enzymes, similarly to the one on the number of soil microorganisms, was relatively small and irregular. It should be noted, however, that grass turf is a system with significant biological inertia, which is much more ecologically stable than the soil under one-year cultivation, and thus, also more slowly reacting to various external factors. Therefore, the response of the microbial population to the applied experimental factors in the second and third year of the use of perennial ryegrass presented in this study was much weaker than in the first year of use [32]. For the same reasons, the effect of the EM-1 preparation, which is only a donor of various microorganisms exposed to a native microbiome, was much weaker (and most often insignificant) than the effects of other combinations, including EM-1 + Ca, whose core activity is to modify habitat conditions (pH, mineral composition, availability of elements, etc.). The other reason for the poorly marked impact of bioconditioners on the number of soil microorganisms and soil enzymatic activity was a relatively high fertility of the soil on which the experiment was established. Meanwhile, it seems that the efficiency of this type of soil enrichment and increasing its activity is the highest on weaker and degraded soils [40].

One of the indicators of soil fertility, which largely determines its physical, chemical and biological properties – and thus the conditions of plant growth and development – is its pH. Changes in pH provide a series of pieces of information on the directions of processes occurring in the soil [41].

**Figure 7:**
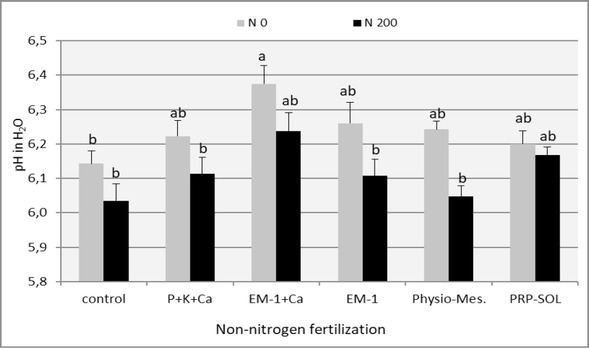
The effect of fertilizer combinations on the soil pH (mean 2008-2009). Means followed by the same letters do not differ significantly (p=0.05)

The influence of the applied experimental combinations on soil pH is presented in Figure 7. This effect was small and in most experimental combinations statistically insignificant. Nevertheless, in all combinations in which bioconditioners were used, a small increase in soil pH was found in relation to the control combinations and the combination of P + K + Ca fertilizers. In the case of PRP-Sol and Physio-Mescal G18 preparations, however, this increase is inadequate to the information by the producers about their strong, beneficial effect on a number of soil parameters (including the elevation and stabilization of its pH). The strongest effect on the soil pH increase was demonstrated by the EM-1 + Ca preparation in combination without nitrogen fertilization. In turn, nitrogen fertilization caused a slight decrease in soil pH in all experimental combinations throughout the entire vegetation period of plants. The acidifying effect of nitrogen fertilization results, inter alia, from the intensification of the alkaline ion leaching process under conditions of an increased concentration of nitrates and from the biological oxidation of ammonium cation, resulting in the release of hydrogen ions [28].

## 4 Conclusion

The traditional mineral fertilization (P+K+Ca) and the use of Physio-Mescal were the most effective in the context of yield and vitality of the perennial ryegrass – regardless of nitrogen fertilization. Physio-Mescal also stimulated soil enzyme activity and the number of soil bacteria. Evaluation of the EM-1 preparation is difficult and ambiguous. Its effect positively modified the addition of calcium. Without this additive the impact of EM-1 on ryegrass yielding, the number of soil microorganisms and phosphatase activity was insignificant compared to the control.

To sum up, it should be noted that the bioconditioners are not an alternative to mineral fertilization as the basic yield factor, but may be a very valuable supplement to this fertilization, which supports the maintenance of the proper biological potential of the soil and its fertility, especially where fertilization with manure or other natural fertilizers is not used.
